# Circumferential approach via dynamic position in OLIF combined with freehand screw pedicle fixation for lumbar tuberculosis requiring multilevel instrumentation: a 3-year retrospective study

**DOI:** 10.1186/s13018-023-03959-3

**Published:** 2023-06-29

**Authors:** Jinyue He, Fei Luo, Qing Fang, Yu Xiang, Jianzhong Xu, Zehua Zhang

**Affiliations:** Department of Orthopedics, Southwest Hospital, Army Medical University, Chongqing, 400038 China

**Keywords:** Lumbar tuberculosis, Oblique lateral interbody fusion, Dynamical position, Freehand pedicle screw fixation, Radiation exposure

## Abstract

**Purpose:**

To advance a modified oblique lumbar interbody fusion (M-OLIF) achieving anterior debridement and posterior freehand instrumentation simultaneously in circumferential approach via dynamic position and compare with traditional combined anterior–posterior surgery (CAPS) in clinical and radiological evaluation.

**Patients and methods:**

Innovative freehand instrumentation in floating position was described. Consecutive patients having undergone surgeries for lumbar tuberculosis from 2017 January to 2019 December had been retrospectively reviewed. Patients with follow-ups for at least 36 months were included and divided into M-OLIF or CAPS group according to surgical methods applied. Outcomes included operation time, estimated blood loss, complication profile for safety evaluation; Vascular Analogue Scale (VAS) and Oswestry Disability Index (ODI) for efficacy evaluation; C-reactive protein and Erythrocyte Sedimentation Rate for tuberculosis activity and recurrence evaluation; X-ray and CT scan for radiological evaluation.

**Results:**

Totally 56 patients had been enrolled in the study (26 for M-OLIF and 30 for CAPS). Compared with CAPS group, M-OLIF group illustrated significantly decreased estimated blood loss, operation time, hospital stay, and less postoperative morbidities. Meanwhile, M-OLIF group showed earlier improvement in VAS in 3 days and ODI in the first month postoperatively, without obvious discrepancy in further follow-ups. The overall screw accuracy in M-OLIF and CAPS group was 93.8% and 92.3% respectively, without significant difference in perforation distribution.

**Conclusion:**

M-OLIF was efficient for lumbar tuberculosis requiring multilevel fixation, with reduced operation time and iatrogenic trauma, earlier clinical improvement compared with traditional combined surgery.

## Introduction

Lumbar had been the main site of extrapulmonary musculoskeletal tuberculosis necessitating surgical treatment if accompanied with progressive bony destruction and abscess formation refractory to medicine treatment [1]. Enormous abscess with severe bony destruction made treatment particularly challenging in how to achieve effective debridement, reconstruction, and instrumentation concurrently. Traditional anterior-only technique emphasized thorough debridement without reliable instrumentation to maintain reconstructed alignment. Meanwhile excessive approach-oriented morbidities had furtherly limited its application [2]. Alternatively, posterior-only surgery gradually became the preferred option for excellent capability in alignment reconstruction and deformity correction. Nevertheless, oblique and inconvenient surgical routine and obstructed visualization significantly limited the capability of effective debridement [3], especially in cases with copious abscess around the anterior-middle column. Therefore, combined anterior–posterior surgery (CAPS) had once been the ideal protocol for reconciling advantages of anterior and posterior approaches [4]. However, excessive iatrogenic trauma and prolonged operative time had put the protocol under increasing controversies [5].

The last decade had witnessed profound progress of lateral extraperitoneal approach in treating degenerative diseases [6]. Meanwhile, innovative application of lateral technique had also been reported in treating lumbar tuberculosis involving single-level fixation with satisfactory clinical results confirmed [7]. Nevertheless, the minimally invasive lateral approach had rarely been recommended in treating lumbar tuberculosis concurrent with severe bony defect requiring multisegmented fixation, as long-segment percutaneous fixation required intraoperative flipping to prone position, which would inevitably lead to staged procedures and prolonged operation time, along with position-oriented morbidities including cage slipping; Meanwhile, excessive radiation exposure had been an increasing concern especially for surgeons performing the surgeries frequently [8]. Therefore, classical lateral approach shouldn’t be regarded as an optimal protocol in treating severe lumber tuberculosis requiring multisegmented fixation until necessary modification was made. In this article, we aimed to advance a modified OLIF technique (M-OLIF): (i) to achieve minimally invasive lateral debridement and posterior instrumentation simultaneously based on dynamic position free from re-antisepsis and re-drape; (ii) achieve freehand posterior pedicle screw fixation with minimal radiation exposure; (iii) provide a reliable standardized minimally invasive lateral protocol suitable for lumbar tuberculosis with severe bony defect warranting multilevel fixation.

## Materials and methods

### Patients

The research protocol was approved by the ethnic committee of our institution in complying with STROCSS Guidance. During a 3-year period from January 2017 to December 2019, consecutive patients diagnosed as lumbar tuberculosis and undergone surgeries had been retrospectively reviewed. Surgery indications included (i) massive abscesses and bony destruction; (ii) segmental instability; (iii) neurological compression; (iv) unendurable back pain refractory to anti-tuberculosis chemotherapy; Inclusion criteria included (i) multilevel fixation involved; (ii) undergone M-OLIF or CAPS surgeries; (iii) follow-ups for at least 36 months. Exclusion criteria included (i) single-level fixation; (ii) severe kyphosis warranting corrective osteotomy; (iii) cauda equina dysfunction or severe radiculopathy necessitating circumferential decompression; (iv) prior intra-abdominal or retroperitoneal surgery or other conditions unsuitable for lateral approach. Consequently, a total of 56 patients had been enrolled in this study, including 26 cases in M-OLIF group and 30 cases in CAPS group.

### Surgical procedure

#### Exposure, debridement, and reconstruction in M-OLIF

**Fig. 1:**
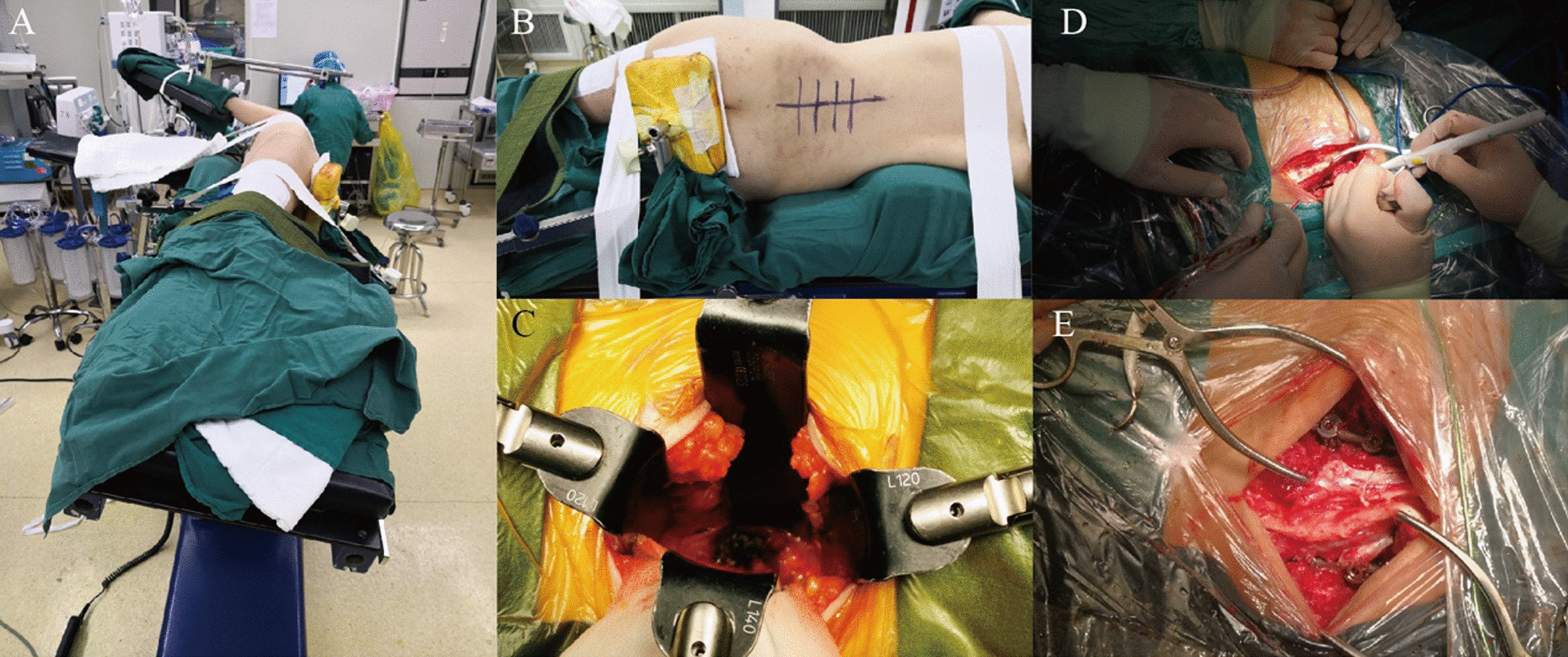
Position manipulation and freehand pedicle screw fixation in M-OLIF. **A** Pre-tilted operation table with patients in true lateral decubitus position; **B** Buffer pads and tapes for adequate postural supporting; **C** lateral access to lesion by retractors; **D**, **E** freehand pedicle screw fixation in dynamic position through Wiltse approach

Patients undergone general anesthesia were secured in lateral position with thorax and pelvis/lower extremities tapped to the table. As a rule, the right decubitus position was preferred to avoid inferior vena cava with neuromonitoring attached. The table was electronically rotated clockwise to the end with the patients readjusted in true lateral relationship with reference to the floor (Fig. 1A). Meanwhile, enough buffer pads should be placed on the ventral and dorsal for adequate postural supporting (Fig. 1B). After the targeted level was localized by X-ray, a 4–6 cm incision anterior to the middle of targeted disc was made. Sequentially, the external oblique, internal oblique, and transversus abdominis were bluntly dissected with fingers along fiber orientation to access underlying retroperitoneal fat and retroperitoneal space. Digital palpation along the transverse process and psoas confirmed ideal orientation. Further ventral palpation would reach the anatomical vacancy between psoas and aorta. Peritoneum were swept ventrally to enlarge the space for surgical field exposure with guiding pin inserted into the vertebra for level identification. During the process above, focus including sequestrum, necrotic discs, pus and other caseous necrosis tissues would be visible and accessible. After expandable retractors were placed sequentially to establish an access corridor and fixed to the table-mounted arm assembly (Fig. 1C), various curettes, rongeurs and scalpels were sequentially used to remove lesion absolutely, followed by copious irrigation with saline solution, hydrogen peroxide, iodophor. Lastly, anterior reconstruction was completed via placement of titanium mesh/cages loaded with autograft particles according to individualized situation.

#### Posterior instrumentation in dynamic floating position in M-OLIF

**Fig. 2:**
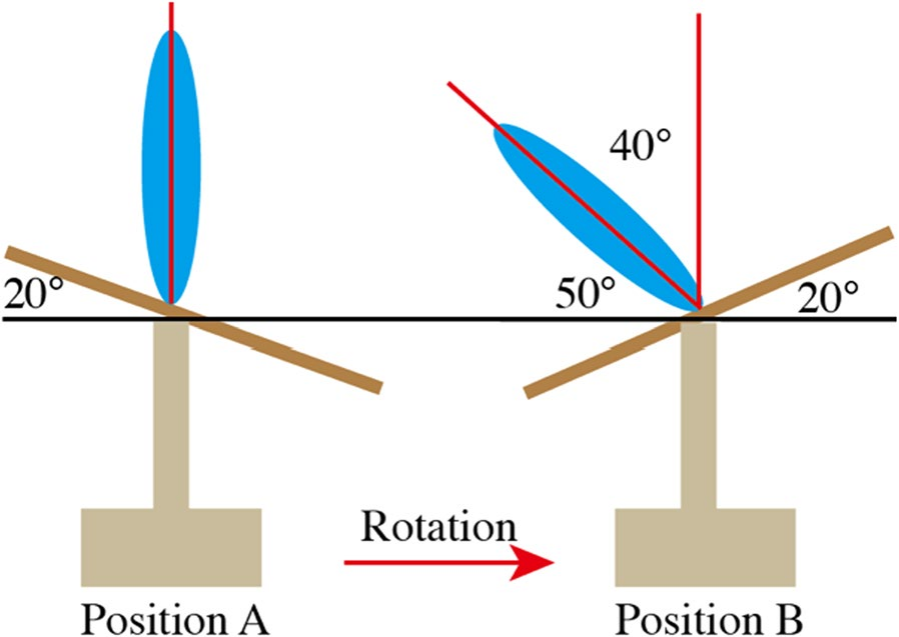
Sketch map of dynamic position. Position A for anterior debridement and reconstruction: pre-tilted operation table (brown) with patients (blue) in true lateral decubitus position with the floor plane(black line); Position B for posterior freehand pedicle screw fixation: Counterclockwise rotated to the end with patient (blue) in an oblique angle of 50 to the floor (black line)

The pre-tilted operation table was counterclockwise rotated electrically to the other end, leading to an oblique angle of 50° of patients in reference to earth floor (Fig. 2). A middle incision was made 1–2 levels above and below diseased vertebrae. After subcutaneous tissue was divided with electrocautery, blunt dissection through Wiltse approach was made in floating position (Fig. 1D) to access the entry points where super articular process (SAP), transverse process, and lamina converged. Instrumentation was performed in direct vision (Fig. 1E) according to SAP-guided freehand technique [9]. In detail, the medial–lateral angle in cannulation should be 5°at L1-2, 20°at L3-4 and 25°at L5 respectively in reference to the sagittal plane of spine. Meanwhile, the trajectory should be vertical to external margin of SAP or lamina as the cranial-caudal orientation. It was worth noting that during process above continuous palpation of resistance was a reliable indicator. Given the presence of anterior-middle column defect, any abnormal feedback during cannulation indicated either pedicle rupture or bone defect, which should be further confirmed by ball-ended feeler before screw placement. Lastly, fluoroscopy was used to evaluate the instrument after all screws were placed.

#### Debridement, reconstruction, and instrumentation in CAPS

Patients undergone general anesthesia we replaced in lateral position. A long oblique incision of 12–18 cm was made with the external oblique, internal oblique, and transversus abdominis dissected by electrotome to access retroperitoneal space. The subsequent debridement and reconstruction were the same as lateral approach in M-OLIF. Then patients were repositioned to standard prone position. A median incision of 8–12 cm was made at the center of diseased vertebrae. Subperiosteal dissection was performed to adequately expose posterior elements to perform instrumentation via SAP-based freehand technique [9].

### Perioperative care

All patients had undergone standardized anti-tuberculosis chemotherapy (isoniazid + rifampicin + pyrazinamide + ethambutol) for more than 4 weeks preoperatively, with ESR and CRP significantly reduced. Postoperatively, focus removed was sent to laboratory immediately for drug-susceptibility tests to guide subsequent anti-tuberculosis chemotherapy, which normally lasted for 18 months.

### Clinical and radiographic evaluation

#### Clinical evaluation

Estimated blood loss, operative time, complication profile, hospital stay, radiological shots per screw were used for clinical safety evaluation; Perioperative Visual Analogue Scale (VAS), Oswestry Disability Index (ODI) for clinical efficacy evaluation; erythrocyte sedimentation rate (ESR) and C-reactive protein (CRP) for evaluating tuberculosis activity and recurrence risk in follow-ups.

#### Radiographic evaluation

Standard anterior–posterior (AP) view and lateral X-ray film were taken perioperatively and in follow-ups (1/3/12/18/36 months) for instrumentation evaluation. Fusion assessment was made after 12 months postoperatively according to Bridwell criteria [10]. In addition, postoperative sliced CT scan was required in all cases to assess debridement and screw accuracy. Upon detecting inaccessible contralateral psoas abscess, additional CT-guided percutaneous drainage catheterization would be performed before discharge from hospital.

All evaluations above were conducted by two independent observers blinded to the study and any dispute would be designated to a third senior surgeon for final confirmation.

### Statistical analysis

Data was presented as mean ± standard deviation for various variation, number and percentage for category variation. Statistics evaluation was made with SPSS 21.0 (IBM, Armonk, NY, USA). Student t-test and Mann–Whitney U was used for continuous variation and Chi-square test for category variation. *P* < 0.05 was regarded significant difference.

## Results

### Demographic data

As shown in Table 1, A total of 56 patients (22 for males and 34 for females) diagnosed as lumbar tuberculosis who had undergone M-OLIF or CAPS during January 2017 and December 2019 had been enrolled in the study (26 for M-OLIF and 30 for CASP). The age ranged 26–69 years with a mean follow-up of 42.1 ± 5.2 months, involving levels from L2–L5. The lesion was distributed in 2 adjacent vertebrae (51 cases) and 3 adjacent vertebrae (5 cases), with 3-vertebra fixation (22 cases), 4-vertebra fixation (29 cases) to 5-vertebra fixation (5 cases). Demographic data between two groups didn’t show significant difference in aging, sex, follow-up, lesion distribution or fixation range (*P* > 0.05).

**Table 1:** Demographic data

	M-OLIF	CAPS	*P*-value
Male/female (cases)	10/16	12/18	0.906
Mean age (years)	49.8 ± 12.2	45.7 ± 11.9	0.252
Mean follow-up (months)	40.8 ± 4.5	43.1 ± 5.6	0.091
Lesion distribution			1.000
2 vertebrae	24	27	
3 vertebrae	2	3	
Fixation range			1.000
3 vertebrae	10	12	
4 vertebrae	14	15	
5 vertebrae	2	3	

*M-OLIF* Modified oblique lumbar interbody fusion; *CAPS* combined anterior posterior surgery

### Clinical evaluation

Clinical safety evaluation: As shown in Table 2, the mean estimated blood loss in M-OLIF group was significantly less than CAPS group (466.9 ± 97.7 vs 1063.0 ± 213.6 ml, *P* < 0.05). Meanwhile, the mean operative time in M-OLIF group was 325.4 ± 52.5 min, obviously less than 412.7 ± 45.8 min in CAPS group (*P* < 0.05). Additionally, M-OLIF group showed significantly shorter hospital stay than CAPS group (9.8 ± 2.2 vs 13.4 ± 2.5 days, *P* < 0.05). The overall complication rate in M-OLIF group was 23.1% including 1 transient hip flexion weakness case and 2 thigh numbness cases, which had been significantly alleviated after 1 week symptomatic therapy. Peritoneal tear had occurred in 2 case and been sutured immediately upon detection; 1 case had presented mild sympathetic trunk-associated symptom which had significantly mitigated after two-week symptomatic treatment. In contrast, 43.3% cases in CAPS group had reported complications consisting of hip flexion weakness in 1 case, transient thigh numbness in 3 cases, peritoneal injury in 6 cases, sympathetic trunk injury in 1 case and poor wound healing in 2 cases. Lastly, similar radiation exposure had been shown between two groups (0.68 vs 0.72 shot per screw).

**Table 2:** Clinical safety evaluation

	M-OLIF	CAPS	*P*-value
Operation time (min)	325.4 ± 52.5	412.7 ± 45.8	0.000
Estimated blood loss (ml)	466.9 ± 97.7	1063.0 ± 213.6	0.000
Hospital stay (days)	9.8 ± 2.2	13.4 ± 2.5	0.000
Fluoroscopy shots/screws	131/194	167/233	
*Complication profile (cases)*			
Hip flexion weakness	1	1	
Transient thigh numbness	2	3	
Vascular injury	0	0	
Peritoneal injury	2	6	
Dural Sac leakage	0	0	
sympathetic trunk injury	1	1	
Poor wound healing	0	2	

Efficacy evaluation: as shown in Table 3, while no marked discrepancy was illustrated in preoperative VAS or ODI between two groups, the mean postoperative VAS score in M-OLIF group was significantly lower than CAPS group in 3 days (2.8 ± 0.4 vs 3.5 ± 0.5, *P* < 0.05), but reduced to a similar level in 1 month postoperatively (1.7 ± 0.5 vs 1.8 ± 0.6, *P* > 0.05), without significant difference in longer follow-ups. Meanwhile, the M-OLIF group showed an improved ODI in the first month postoperatively compared with CAPS group (25.5 ± 4.8 vs 31.5 ± 4.7) (*P* < 0.05), without obvious distinction at the last follow-up. As for laboratory indicators, the mean preoperative CRP and ESR were 46.3 ± 9.1 mg/L and 57.3 ± 12.9 mm/h in M-OLIF group, without significant difference in CAPS group (44.1 ± 18.0 mg/L and 54.5 ± 17.0 mm/h) (*P* > 0.05). Notably, the CRP and ESR in M-OLIF group was increased to 69.8 ± 24.3 mg/L and 68.5 ± 20.1 mm/h in 3 days after surgery, significantly lower than CAPS group (94.8 ± 19.1 mg/L and 119.6 ± 30.4 mm/h) (*P* < 0.05). Both indicators in two groups had decreased to a similar level in 1 month without obvious distinction at the last follow-up.

**Table 3:** Clinical efficacy and recurrence risk evaluation

Patient-reported				Laboratory test			
	M-OLIF	CAPS	*P*-value		M-OLIF	CAPS	*P*-value
*VAS*				*CRP*			
Pre-op	6.2 ± 0.7	6.0 ± 0.6	0.301	Pre-op	46.3 ± 9.1	44.1 ± 18.0	0.899
3 days post-op	2.8 ± 0.4	3.5 ± 0.5	0.000	3 days post-op	69.8 ± 24.3	94.8 ± 19.1	0.000
1 month post-op	1.7 ± 0.5	1.8 ± 0.6	0.791	1 month post-op	11.8 ± 3.9	13.2 ± 7.7	0.777
3 months post-op	0.8 ± 0.4	0.9 ± 0.5	0.421	3 months post-op	7.1 ± 2.0	7.5 ± 2.4	0.638
18 months post-op	0.7 ± 0.5	0.8 ± 0.6	0.751	18 months post-op	5.5 ± 1.3	5.7 ± 1.3	0.715
Last follow-up	0.6 ± 0.4	0.7 ± 0.5	0.584	Last follow-up	5.8 ± 1.6	5.7 ± 1.1	0.949
*ODI*				*ESR*			
Pre-op	50.2 ± 6.6	53.2 ± 8.4	0.155	Pre-op	57.3 ± 12.9	54.5 ± 17.0	0.506
1 month post-op	25.5 ± 4.8	31.5 ± 4.7	0.000	3 days post-op	68.5 ± 20.1	119.6 ± 30.4	0.000
3 months post-op	17.5 ± 4.3	16.7 ± 4.4	0.585	1 month post-op	14.2 ± 3.4	13.4 ± 3.3	0.362
18 months post-op	12.9 ± 1.9	13.1 ± 2.3	0.858	3 months post-op	8.2 ± 2.1	8.6 ± 2.6	0.439
Last follow-up	9.4 ± 1.9	10.3 ± 1.7	0.147	18 months post-op	5.8 ± 1.7	6.4 ± 1.8	0.208
				Last follow-up	5.4 ± 1.3	5.6 ± 1.5	0.594

*VAS* Vascular analogue scale; *ODI* Oswestry disability index; *CRP* C-reactive protein; *ESR* Erythrocyte sedimentation rate

### Radiological evaluation

As shown in Table 4, postoperative CT scan showed the overall screw accuracy was 93.8% in M-OLIF group, comparable with 92.3% in CAPS groups. Screw perforation in both groups was distributed in medial, lateral and superior orientation, without significant difference in orientation distribution between two groups (*P* = 0.649). No medial perforation was shown to exceed 4 mm, with limited risk of neurological risk. According to Bridwell’s criteria on fusion assessment via X-ray [10], 92.3% (24/26) cases in M-OLIF had attained grade I fusion while 2 cases had been rated as grade II. Comparably, 90.0% (27/30) cases in CAPS group had been rated grade I with the remaining 3 cases as grade II. Through the whole follow-up, no drug-resistant tuberculosis or recurrence had been confirmed in any group.

**Table 4:** Radiographic evaluation

	M-OLIF	CAPS	*P*-value
Screw placement	194	233	
Screw perforation	12	18	0.649
Medial	6	11	
Lateral	4	6	
Superior	2	1	
Inferior	0	0	
Fusion evaluation			1.000
Grade I	24	27	
Grade II	2	3	
Grade III	0	0	
Grade IV	0	0	
Tuberculosis recurrence	0	0	
Instrument failure	0	0	

### A representative case

**Fig. 3:**
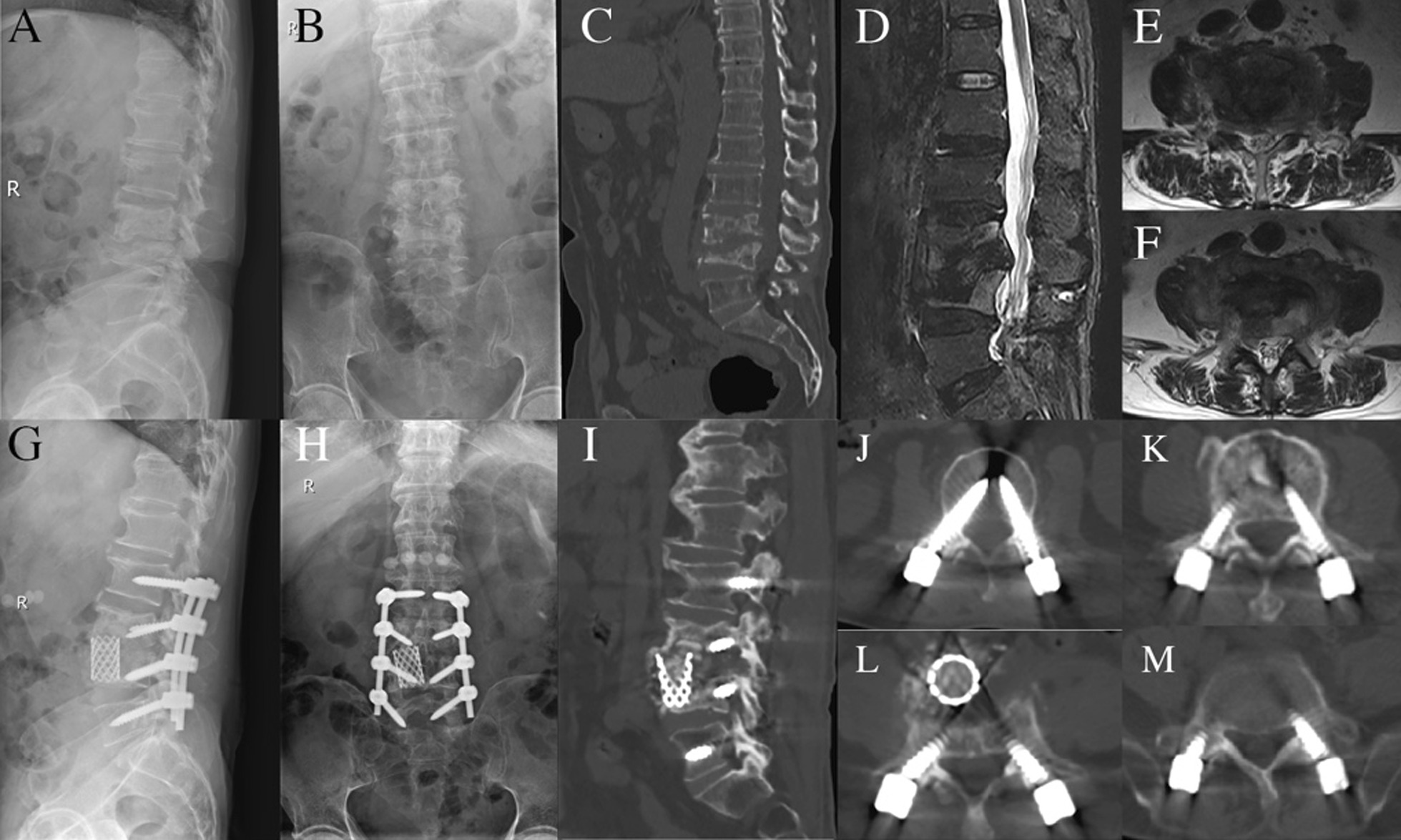
Radiographic images of a representative case diagnosed as L3, 4 tuberculosis. **A**, **B** preoperative X-ray in lateral and AP view. **C** preoperative CT in lateral view showing the obvious bony destruction in L3, 4; **D**, **E**, **F** MRI in lateral and cross-sectional view showing obvious abscess formation and severe bony defect; **G**, **H** postoperative X-ray in lateral and AP view showing reconstruction with a titanium mesh in L3/4 and multisegmented fixation from L2–5; **I**, **J**, **K**, **L**, **M** postoperative CT scan in lateral view and cross-sectional views showing the mesh and screws in place. *AP* Anterior–posterior

The perioperative radiography of a representative case diagnosed as L3/4 tuberculosis having undergone M-OLIF surgery was shown in Fig. 3.

## Discussion

### Deficiency in current treatment

Lumbar tuberculosis had accounted for most osteoarticular tuberculosis with a high prevalence in developing countries secondary to socioeconomical condition and immune-compromising diseases [11]. Despite of profound progress in medicine and surgery techniques, the optimal protocol still remained controversy in how to achieve effectively thorough debridement, rigid reconstruction and instrumentation synchronously in treating tuberculous spondylitis with severe abscess and bony deficiency. Traditional anterior-only surgery had been the first solution due to excellent visualization during debridement and reconstruction [12]. Nevertheless, excessive approach-oriented morbidities combined with inadequate deformity correction and alignment maintenance had limited its wider application. As an alternative, posterior-only approach was increasingly performed due to circumferential neurological decompression, effective deformity correction and reliable three-column fixation. However, oblique maneuvering routine combined with obstructed vision greatly limited its capability in thorough debriding. Meanwhile multisegmented reconstruction with titanium mesh in posterior approach was also accompanied with significant risk of neurological injuries. Therefore, combined anterior and posterior surgery uniting advantages of two approaches above had once been accepted as a promising alternative in treating severe lumbar tuberculosis requiring multisegmented fixation. Nevertheless, traditional combined technique was inevitably accompanied with excessive approach-oriented morbidities and prolonged operation time [13], which were extremely challenging to patients with poor physical condition. Recent years had witnessed innovative application of minimally invasive lateral retroperitoneal approach in treating single-level lumbar tuberculosis with satisfactory results confirmed. Nevertheless, to our knowledge, few studies had recommended minimally invasive lateral technique in treating lumbar tuberculosis with severe bony defect requiring multisegmented fixation, which may be ascribed to reasons as bellow: (i) Position-related: Typical posterior percutaneous fixation was generally performed with patients flipped to prone position, necessitating re-antisepsis and re-drape, leading to increased operation time and position-oriented risk; (ii) Radiation-related: Percutaneous instrumentation in multisegmented fixation would inevitably result in excessive radiation exposure. Aiming at drawbacks above, our team had attempted to perform lateral debridement and posterior freehand instrumentation concurrently via dynamic position by utilizing an electronic bed, achieving innovative unification of lateral and posterior approach without intraoperative reposition and radiation exposure.

### The innovation and advantages of M-OLIF technique based on dynamic position

#### Reduced iatrogenic injury

Different from traditional combined surgery with a large oblique incision resulting in enhanced risk of vascular, visceral, and neurological morbidities [14, 15], M-OLIF technique accessed retroperitoneal space via a small incision which barely accommodated an adjustable retractor system. Meanwhile, abdominal muscular complex was bluntly dissected along muscle orientation via fingers rather than sharp dividing by electrotome, significantly limiting intraoperative bleeding in exposure. In addition, Posterior instrumentation was performed via Wiltse approach with limited injury to musculoligamentous complex. Consequently, due to effective damage control in debridement and instrumentation, the mean blood loss in M-OLIF group had reduced to 466.9 ± 97.7 ml, significantly less than 1063 ± 213.6 ml in CAPS group. Meanwhile, the inflammatory indicators (CRP and ESR) also demonstrated a lower level in M-OLIF group in 3 days postoperatively, corresponding with the result in estimated blood loss above, indicating less inflammatory response secondary to less iatrogenic trauma. Furthermore, in terms of pain evaluation, the mean VAS score in M-OLIF group was significantly less than CAPS group in 3 days postoperatively but reduced to a similar level after 1 month, illustrating improved pain alleviation at early stage. Meanwhile, the ODI in M-OLIF group also presented an improved result in the first month. Earlier improvement in VAS and ODI suggested less suffering for patients conducible to early off-bed mobilization, which would promote further rehabilitation in turn. Lastly, due to the minimally invasive advantages in debridement and instrumentation, overall complication rate in M-OLIF group was also obviously decreased compared with CAPS group, especially in approach-related peritoneal violations. Analyzing the difference, overexposure of peritoneal in traditional combined surgery did increase the risk of peritoneal breakage despite of discreet precautions. Though immediate saturation was performed upon detection, there existed risk of disastrous complications including tuberculosis peritonitis.

#### Obviation of position flipping and radiation exposure

The obviation of intraoperative position flipping was another unique innovation of M-OLIF technique over CAPS surgery. As rigid long-term fixation was critical in maintaining reconstructed alignment in presence of severe bony defect while affected vertebrae failed to provide adequate purchase and rigidity, different strategies had been advanced. One strategy advocated standardized posterior percutaneous instrumentation in prone position, which had been widely performed worldwide in single-level degenerative cases. However, excessive radiation exposure involved had become a growing concern given the potential hazards to surgeons. Alternatively, freehand techniques had remained the preferred option for many surgeons due to adequate accuracy, reliable safety, and streamline procedure with satisfactory clinical and radiological results reported [16], which were attained at the cost of excessive approach-oriented complications. Moreover, regardless of freehand technique or fluoroscopy-guided technique, patient must be repositioned from lateral position into prone position leading to re-antisepsis and re-drape [17–19], inevitably resulting in staged procedure and prolonged operative time. As a resolution, M-OLIF technique utilized the electric operation bed to achieve position transformation dynamically from lateral position to a “floating position” seamlessly. The “floating position” was a semi-prone position between lateral and prone position, constituting a familiar maneuvering environment similar with prone posture, which was complying with surgeons’ habits. As re-antisepsis and re-drape was obviated during the position transformation, the whole operative time was significantly reduced compared with traditional combined surgery (325.4 ± 52.5 vs 412.7 ± 45.8 min). In addition, in traditional combined surgery patients must be flipped to prone position manually for posterior instrumentation, carrying with it potential risk of graft slipping during reposition. In contrast, patients undergone M-OLIF surgery was repositioned via an electric operation bed smoothly by a 40 angle instead of being manually turned over by a 90 angle, greatly reducing risk of position-oriented morbidities. Furthermore, after posterior instrumentation, patients could be rotated reversely again to lateral position to confirm the inserted cage or titanium mesh in place with rigidity, which was infeasible in traditional combined surgeries. Meanwhile, secondary to the convenience in performing freehand instrumentation in floating position, extra radiation wasn’t necessary, leading to a similar radiation level with CAPS group (0.68 vs 0.72 shot per screw), and we hadn’t detected significant difference in screw accuracy between two groups, indicating reliable feasibility in performing freehand fixation in floating position without compromising accuracy and safety.

### Tips for M-OLIF technique

#### Concerns for indications

It should be emphasized that severe lumbar tuberculosis necessitating multisegmented fixation was the main indication to the present technique. Notably, due to the innovation of combining the minimal invasiveness advantage of lateral approach with the convenience of posterior instrumentation free from position flipping, the present technique was also feasible in treating degenerative diseases necessitating multisegmented fusion. In fact, we had performed the technique in treating degenerative disease involving multilevel fusions with satisfactory clinical and radiological results confirmed. Multisegmented moderate spondylolisthesis or stenosis with low back pain as the chief complaint without severe radiculopathy was the main indication. The obviation of position flipping without fluoroscopy or navigation guidance was the main advantages over traditional staged percutaneous fixation. Nevertheless, with respect to single-level cases, simultaneous lateral debridement, reconstruction, and posterior fixation in single position was usually the optimal alternative instead. Secondly, severe radiopathology or cauda equina symptom weren’t indications for M-OLIF technique resulting from the lack of direct circumferential neurological decompression. Additionally, despite dynamic position allowed manipulations to posterior column in direct visualization including resection of articular process and lamina to achieve SPO ostectomy, more aggressive osteotomy correction wasn’t as feasible in dynamic position as in prone position. Hence, the present technique wasn’t suitable for cases with severe deformity. Nevertheless, alongside the development of cage material, deformity correction via anterior approach with expandable cages was regarded as an promising alternative covering the inadequate ability of anterior correction. Other approach-related contraindications included (i) prior vascular reconstructive surgery; (ii) prior intra-abdominal or retroperitoneal surgery; (iii) history of severe pelvic inflammatory disease [20].

#### Concerns for instrumentation

As bone defect was common presence in severe lumbar tuberculosis, meticulous palpation in cannulation was of utmost importance to confirm the right trajectory with enough purchase. Generally, severe bone defect or osteoporosis would oblige the fixation range to extend one or two level beyond diseased vertebrae. Therefore, short-segment fusion combined with long-segment fixation was a common protocol in our practice, followed by staged fixation removal after at least 1 year. Lastly, it had to be admitted a learning curve existed before getting accustomed to freehand instrumentation in floating position. In our clinical practice, 3–5 operations were required to familiarize freehand fixation in dynamic position for surgeons experienced in freehand technique, without extra fluoroscopy assistance warranted. Prior experience of freehand fixation in prone position would smooth the learning curve and shorten the time required.

### Limitation of the study

Though the study had provided preliminary evidence of advantages of the M-OLIF technique over traditional combined surgery in treating lumbar tuberculosis requiring multisegmented fixation, it had to be noted that the study was a retrospective study conducted in a single center. A prospective multi-center study with a larger sample was warranted in future to further evaluate the technique’s reliability and validity.

## Conclusion

M-OLIF based on dynamic position was an efficient minimally invasive technique in treating lumbar tuberculosis requiring multilevel fixation, with reduced operative time, decreased iatrogenic trauma and earlier clinical improvement compared with traditional combined anterior–posterior surgery.

## Data Availability

All the data and materials have been available and haven’t been under consideration prior to the submission.
